# Comprehensive analysis of copper-metabolism-related genes about prognosis and immune microenvironment in osteosarcoma

**DOI:** 10.1038/s41598-023-42053-w

**Published:** 2023-09-12

**Authors:** Zili Lin, Yizhe He, Ziyi Wu, Yuhao Yuan, Xiangyao Li, Wei Luo

**Affiliations:** 1 Department of Orthopaedics, Xiangya Hospital, Central South University, Changsha, 410008 Hunan People’s Republic of China; 2 Department of Orthopaedics, The Second Xiangya Hospital, Central South University, Changsha, 410011 Hunan People’s Republic of China; 3 National Clinical Research Center for Geriatric Disorders, Xiangya Hospital, Changsha, China

**Keywords:** Bone cancer, Sarcoma

## Abstract

Despite being significant in various diseases, including cancers, the impact of copper metabolism on osteosarcoma (OS) remains largely unexplored. This study aimed to use bioinformatics analyses to identify a reliable copper metabolism signature that could improve OS patient prognosis prediction, immune landscape understanding, and drug sensitivity. Through nonnegative matrix factorization (NMF) clustering, we revealed distinct prognosis-associated clusters of OS patients based on copper metabolism-related genes (CMRGs), showing differential gene expression linked to immune processes. The risk model, comprising 13 prognostic CMRGs, was established using least absolute shrinkage and selection operator (LASSO) Cox regression, closely associated with the OS microenvironment's immune situation and drug sensitivity. Furthermore, we developed an integrated nomogram, combining the risk score and clinical traits to quantitatively predict OS patient prognosis. The calibration plot, timeROC, and timeROC analyses demonstrated its predictable accuracy and clinical usefulness. Finally, we identified three independent prognostic signatures for OS patients: COX11, AP1B1, and ABCB6. This study confirmed the involvement of CMRGs in OS patient prognosis, immune processes, and drug sensitivity, suggesting their potential as promising prognostic signatures and therapeutic targets for OS.

## Introduction

Osteosarcoma (OS), the most prevalent primary malignant bone tumor, predominantly affects children and adolescents, characterized by intratumoral osteogenesis^1^. Significant advancements in OS treatments, such as chemotherapy, neoadjuvant chemotherapy, and radiotherapy, have substantially improved long-term survival rates, leading to a noteworthy increase in the 5-year survival rate, reaching 70%^2^. However, OS metastasis remains a formidable therapeutic challenge, with a distressing 5-year survival rate of only approximately 30% for patients with lung metastases^3^. The genomic instability and complexity of OS have been unequivocally demonstrated to compromise therapeutic efficacy, resulting in unfavorable clinical outcomes for OS patients^4^. Consequently, an urgent need exists to enhance early detection, treatment efficacy, and prognosis prediction for OS by delving deeper into its molecular genetics. In recent years, the continual progress and widespread adoption of high-throughput sequencing technologies and advanced bioinformatics approaches have facilitated a comprehensive investigation of the molecular mechanisms underpinning the biological and pathological processes associated with OS. Notably, the identification and utilization of genes involved in critical pathways such as ferroptosis, apoptosis, and autophagy have contributed to the construction of robust prognostic models, empowering more precise and reliable prognosis predictions for OS patients. Moreover, the development of sophisticated immune infiltration algorithms has provided invaluable insights into the intricate tumor microenvironment, offering researchers novel perspectives on designing targeted immunotherapies for various malignancies, including OS. As a result, the integration of bioinformatics methodologies in OS research holds tremendous potential for unveiling emerging therapeutic directions, which could significantly impact the landscape of OS treatment strategies in the future.

Since the discovery of cuproptosis, a novel form of cell death, the role of copper metabolism in the pathogenesis and progression of diverse diseases has attracted significant attention^5^. Copper, as an essential mineral nutrient, plays a crucial role in various biological processes, including mitochondrial respiration, antioxidation, detoxification, and iron absorption^6^. However, emerging studies have indicated that dysregulated copper homeostasis may be associated with several human diseases^7,8^. Notably, in the context of cancer, disruptions in copper metabolism have been implicated in promoting disease progression by facilitating cancer cell proliferation, growth, angiogenesis, and metastasis^8–12^. There is compelling evidence linking copper dysregulation to the onset and advancement of hepatocellular carcinoma, pancreatic cancer, prostate cancer, and influencing the stage and progression of colorectal and breast cancer^13–18^. Moreover, accumulating evidence suggests that copper actively contributes to the regulation of proangiogenic and angiogenic factors, exerting significant influence on biological processes and pathological advancements involved in the development and progression of cancers^19–21^. Furthermore, copper-dependent molecules have been identified as key players in the occurrence and advancement of various cancers^22–24^. Given these significant findings, manipulating copper metabolism and its associated molecules presents a potential and promising therapeutic avenue for cancer treatment. By targeting the pathways associated with copper metabolism, it may be feasible to modulate disease progression and enhance patient outcomes. Ongoing research in this domain holds the potential for the development of innovative therapeutic strategies that could transform the landscape of cancer treatment in the future.

In the present studies, we performed the least absolute shrinkage and selection operator (LASSO) Cox regression analysis to construct a risk model associated with copper metabolism and established an integrated nomogram to quantitatively predict the prognosis of OS patients. Meantime, we explored the relationship between risk scores and OS immune microenvironment and drug sensitivity. Eventually, through multivariate Cox regression and IHC, we identified COX11, AP1B1, and ABCB6 as three independent prognostic signatures of OS patients, which may provide new targets for OS treatment.

## Materials and methods

### Data acquisition and processing

We downloaded the RNA sequencing data and clinical information of the training datasets TARGET-OS including 85 OS patients from the website UCSC Xena (https://xena.ucsc.edu)^25^. Additionally, the RNA sequencing data and clinical information of the validated datasets GSE21257 including 50 OS patients were downloaded from the Gene Expression Omnibus (GEO) database (https://www.ncbi.nlm.nih.gov/geo)^26,27^. The copper-metabolism-related genes (CMRGs) were downloaded from Kyoto Encyclopedia of Genes and Genomes (KEGG) website^28^. Background correction and normalization were performed for the gene expression matrix of GEO and Xena website in the R version 4.2.1 software. With reference to the merger of two data sets, we firstly used R software package inSilicoMerging to merge data sets^29^ and then utilized the method introduced by Johnson WE et al. to remove the batch effect^30^ (Figure S1). Our acquisition and application of all these data was in accordance with the policies and guidelines of the Xena and GEO databases.

### Unsupervised clustering of CMRGs

The nonnegative matrix factorization (NMF) clustering was applied to separate patients into distinct molecular subtypes based on the CMRGs via "NMF" package. The Kaplan–Meier curves were performed for distinct clusters, and clusters with prognostic curves close to each other were merged. Then, the differentially expressed analysis between the new clusters were calculated using the "limma" package, and genes (adjusted p value < 0.05 and foldchange > 2) are defined as differentially expressed genes (DEGs). Additionally, to explore the potential mechanism of the copper metabolism on OS, the functional enrichment including Gene ontology (GO: GO_biological process, GO_cellular component, GO_molecular function)^31^ and KEGG analysis were performed for DEGs. Moreover, for the further validation of the functional enrichment results of DEGs, Over-representation Analysis (ORA)^32^, a kind of Gene Set Enrichment Analysis (GSEA)^33^, was performed to explore the KEGG pathways associated with the clusters. The adjusted p value = 0.05 was set as cut-off of both the functional enrichment of DEGs and GSEA analyses. Additionally, the Estimation of Stromal and Immune cells in Malignant Tumor tissues using Expression data (ESTIMATE)^34^ algorithm was implemented to determine the stromal score, immune score, estimate score, and tumor purity of distinct molecular subgroups.

### Screening of prognosis-related CMRGs and construction of the prognostic model

The univariate cox regression were performed for all the CMRGs and the CMRGs with the p value less than 0.05 were identified as the prognosis-related CMRGs. Then, to eliminate the effect of overfitting, LASSO Cox regression^35^ was performed to construct the prognostic model and the “lambda.min” was used as the lambda value. Additionally, GSE21257 dataset was considered as the validated dataset. The risk model can be expressed as follows:

$$_{.} {\text{Risk}}\,{\text{score}} = \sum {\text{Coefficient}}_{{{\text{mRNAi}}}} \times {\text{Expression}}_{{{\text{mRNAi}}{.}}}$$

### Functional enrichment and immune infiltration analysis

The OS patients were separated into high-risk and low-risk group based on the median risk score. Additionally, the DEGs between high-risk and low-risk group were screened out and the functional enrichment including GO and KEGG analyses were carried out, with the adjusted p value = 0.05 being cut-off. Furthermore, the immune infiltration analyses including Cell type identification by estimating relative subsets of RNA transcripts (CIBERSORT)^36^ and ESTIMATE algorithm were implemented to analyze the proportion of immune cells. Meantime, the analyses of cancer immunity cycles and immunotherapy-predicted pathways were carried out to depict the immune landscape of OS^37,38^.

### Construction and validation of the integrated nomogram

The multivariate cox regression was performed for the risk score and other clinical traits including age, gender, and metastasis and the variables with p value less than 0.05 were included in the nomogram. Subsequently, the calibration curve, timeROC curve, and timeDCA curve of the nomogram were depicted to validate the effect of the nomogram.

### Drug sensitivity analysis

The "limma", "ggpubr", and "oncoPredict" packages were utilized to perform drug sensitivity analysis between high- and low-risk group to screen potential therapeutic drugs for OS, with adjusted p value < 0.0005 as the screening criterion.

### Immunohistochemical analysis

OS tissues and adjacent normal tissues were derived from patients undergoing knee arthroplasty in the Orthopaedics Department of Xiangya Hospital. The Ethics Committee of Xiangya Hospital of Central South University approved this study, and informed consent was obtained from all the participants or their legal guardians. All operations involving human organizations or tissues were performed in accordance with the Declaration of Helsinki. The OS tissues and adjacent normal tissues were fixed, embedded and sectioned. Subsequently, deparaffinization and dehydration were performed for each slide. After antigen reparation, the slides were incubated with 3% hydrogen peroxide solution at room temperature for 10 min to block endogenous peroxidase activity. After rinsing with PBS, the slides were incubated for 1 h at room temperature with the goat serum (ZLI-9022, ZSGB-Bio, China). Then, the slides were hatched with COX11 primary antibody (11498-1-AP, proteintech, China), AP1B1 (R160051, ZENBIO, China) and ABCB6 primary antibody (R121438, ZENBIO, China) which were diluted into 1:100, at room temperature overnight. After rinsing in PBS for three cycles 5 min/times, the slides were hatched with antibody booster and anti-rabbit secondary antibody (PV-9000, ZSGB-Bio, China) for 20 min at room temperature, respectively. Finally, the signals of sections were developed using 3,3′-diaminobenzidine tetrahydrochloride, and all slides were stained with hematoxylin.

### Statistical analysis

Statistical tests were performed using R software (version 4.2.1). Continuous data were expressed as the mean and standard deviation, while categorical data were expressed as count and percentage. Univariate, lasso and multivariate Cox analysis were performed to identify independent prognostic factors and construct an integrated nomogram including predictable clinical traitors and risk score. The performance and clinical usefulness of model were assessed by calibration curve, timeROC and timeDCA. All tests were two-sided. The statistical significance was shown as follows: p < 0.05 (*), p < 0.01 (**), p < 0.001 (***).

### Ethics approval and consent to participate

This study did not involve any human samples and was approved by the Ethics Committee of Xiangya Hospital of Central South University.

## Results

### Identification of CMRGs clusters and their correlation with biological functions in OS

**Figure 1:**
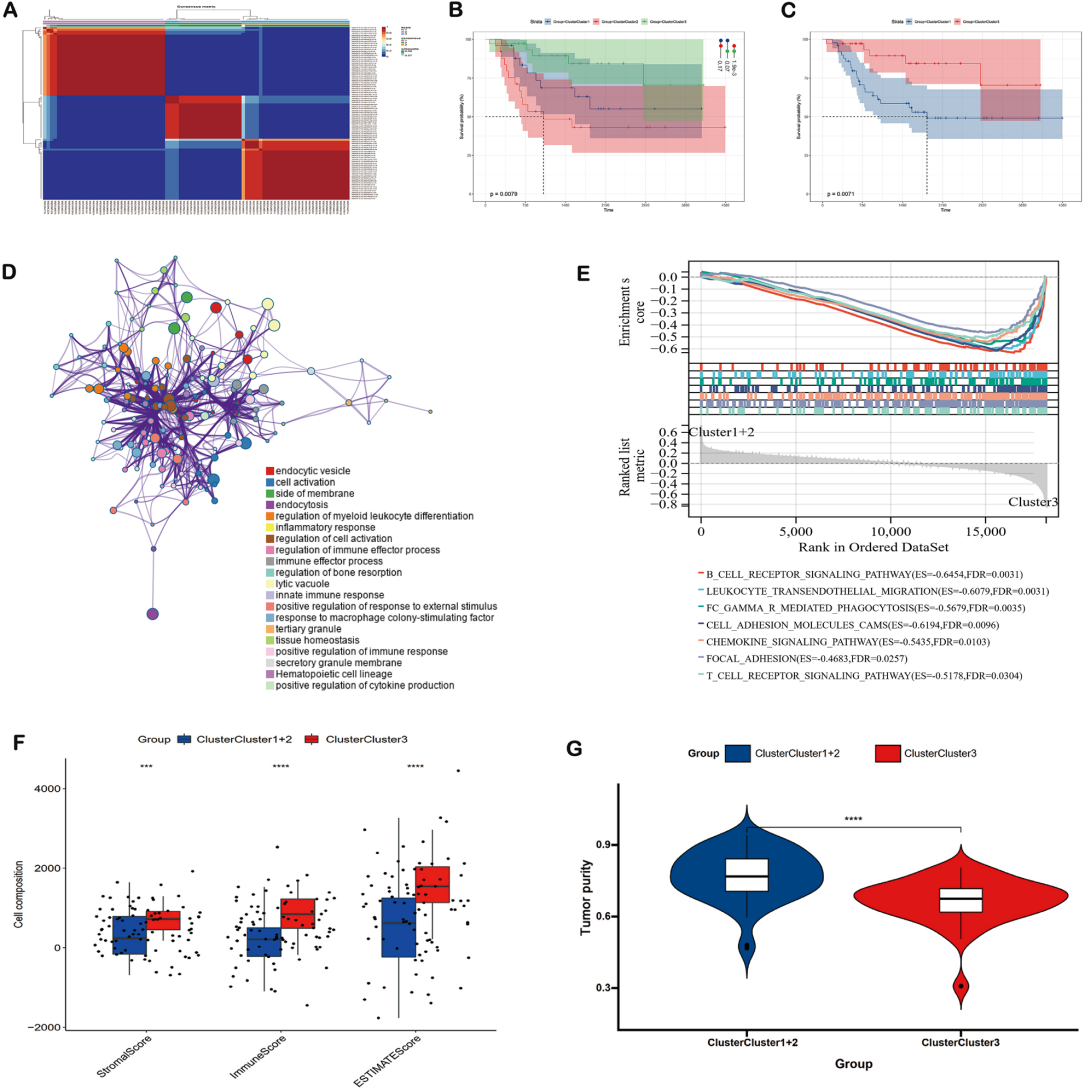
The role of copper metabolism in osteosarcoma. (**A**) Clustering based on CMRGs (**B**,**C**) Prognosis of different clusters; (**D**––**F**) volcano plot, GO, and KEGG analyses of DEGs between different clusters. (**G**) GSEA analysis of different clusters. (**H**,**I**) ESTIMATE analysis of different clusters. *CMRGs* copper-metabolism-related genes, *DEGs* differentially expressed genes.

There were 118 CMRGs downloaded from the KEGG website and 107 CMRGs were retained after eliminating the genes that are not expressed in 50% TARGET-OS samples (Table S1). To explore the role of CMRGs in OS, we used an NMF algorithm to classify the OS patients according to the expression of the 107 CMRGs and the results showed dividing into 3 clusters as the optimal clustering method (Figure S2): Cluster 1 (n = 25), Cluster 2 (n = 25), and Cluster 3 (n = 35) (Fig. 1A). Subsequently, the Kaplan–Meier curve was performed for these clusters and survival analysis revealed that the patients in cluster 2 suffered the worst prognosis, while cluster 3 patients had the best prognosis (Fig. 1B). Then, cluster 1 and 2 were merged into a group due to the proximity of their survival curve and the prognosis of patients in cluster 3 is also significant better than that of the merged cluster (p value = 0.0071) (Fig. 1C). Subsequently, the DEA was performed for the cluster 3 and the merged cluster, and the genes with |log2Foldchange|> 1 and adjusted p value < 0.05 were identified as DEGs (Table S2). Then, the functional enrichment analyses were performed for the DEGs and the top 20 results were mostly enriched in the immune-related pathways including endocytic vesicle, endocytosis, regulation of myeloid leukocyte differentiation, inflammatory response, regulation of immune effector process, and immune effector process (Fig. 1D, Table S3). Furthermore, the result of GSEA were enriched in B cell receptor signaling pathway, leukocyte transendothelial migration, FC gamma R mediated phagocytosis, cell adhesion molecules, chemokine signaling pathway, focal adhesion, and T cell receptor signaling pathway (Fig. 1E). Considering the focus of the functional enrichment on immune related pathways, we performed immune infiltration analysis, and ESTIMATE algorithm showed that compared with the merged cluster, cluster 3 exhibited a higher situation in stromalscore, immunescore, and estimatescore but a lower situation in tumor purity (Fig. 1F,G). Therefore, copper metabolism may play an importance in the development of OS and related to the immune process of the tumor microenvironment.

### Identification of the prognosis-related CMRGs and construction of the prognostic model

To identify the prognosis-related CMRGs, the univariate cox regression were performed for all CMRGs and 16 CMRGs with the p value less than 0.05 were identified as the prognosis-related CMRGs (Table S4) and included in the prognostic model: SNCB, AP1B1, LOX, ABCB6, MT1G, LOXL1, AOC3, COG2, LOXL4, COX17, COX11, CDK1, PRNP, S100A13, MT1H, and MT1A (Table 1). Subsequently, we utilized LASSO regression to narrow the prognostic model (Figure S2). Finally, the prognostic model contained 13 genes and could be expressed as (Figures S3–4): Riskscore = 0.41 × SNCB − 0.68 × AP1B1 + 0.42 × LOX + 0.63 × ABCB6 + 0.03 × MT1G + 0.36 × AOC3-0.14 × LOXL4 + 0.02 × COX17 − 0.84 × COX11 + 0.17 × CDK1 + 0.14 × S100A13 + 0.12 × MT1H + 0.35 × MT1A.

**Table 1:** Summary of descriptive characteristics of the included articles (n = 8).

Sig_genes	Full name	Category	Gene card ID	P value	HR (95% CI for HR)
CFH	Complement Factor H	Protein coding	GC01P196621	0.00063	0.54 (0.38–0.77)
SLC16A8	Solute Carrier Family 16 Member 8	Protein coding	GC22M067673	0.0071	1.50 (1.10–2.00)
GATA1	Lysyl Oxidase	Protein coding	GC05M122063	0.0016	1.90 (1.30–2.80)
ABCB6	ATP Binding Cassette Subfamily B Member 6	Protein coding	GC02M219209	0.0018	2.80 (1.50–5.30)
MT1G	Metallothionein 1G	Protein coding	GC16M056666	0.035	1.30 (1.00–1.60)
LOXL1	Lysyl Oxidase Like 1	Protein coding	GC15P073925	0.0056	0.72 (0.57–0.91)
AOC3	Amine Oxidase Copper Containing 3	Protein coding	GC17P042851	0.0024	1.90 (1.30–2.90)
COG2	Component Of Oligomeric Golgi Complex 2	Protein coding	GC01P230642	0.04	2.90 (1.00–8.00)
LOXL4	Lysyl Oxidase Like 4	Protein coding	GC10M098247	0.0092	1.50 (1.10–2.00)
COX17	Cytochrome C Oxidase Copper Chaperone COX17	Protein coding	GC03M119654	0.035	1.60 (1.00–2.60)
COX11	Cytochrome C Oxidase Copper Chaperone COX11	Protein coding	GC17M054951	0.0099	0.24 (0.08–0.71)
CDK1	Cyclin Dependent Kinase 1	Protein coding	GC10P060772	0.032	1.80 (1.10–3.10)
PRNP	Prion Protein	Protein coding	GC20P004686	0.011	0.49 (0.28–0.85)
S100A13	S100 Calcium Binding Protein A13	Protein coding	GC01M153618	0.00097	2.20 (1.40–3.50)
MT1H	Metallothionein 1H	Protein coding	GC16P057086	0.011	1.50 (1.10–2.10)
MT1A	Metallothionein 1A	Protein coding	GC16P056638	0.00023	1.60 (1.20–2.10)

### Risk score was associated with the prognosis of OS patients and immune infiltration

**Figure 2:**
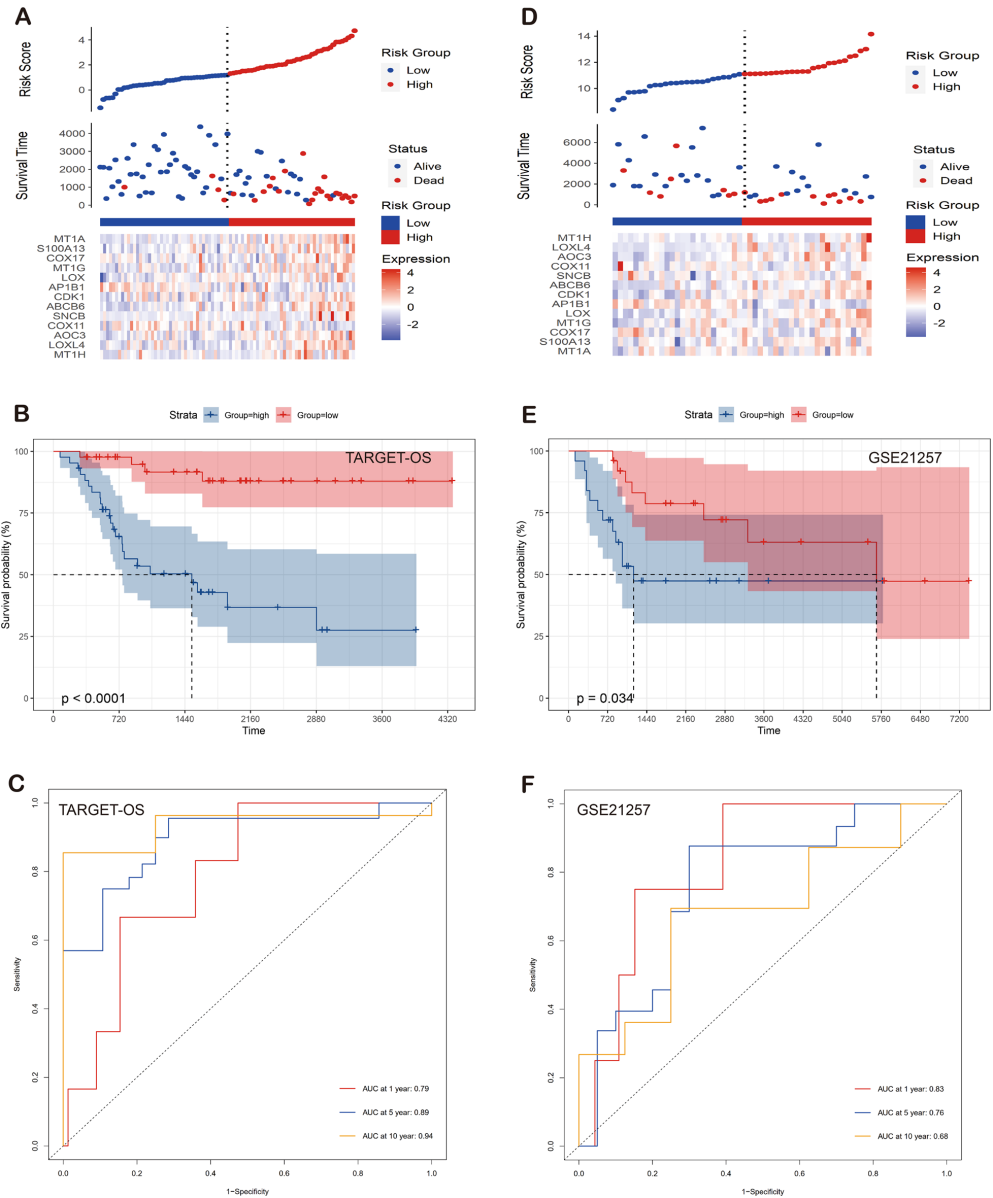
Construction and validation of the prognosis risk model. (**A**–**F**) Prognostic value in training and testing cohort.

**Figure 3:**
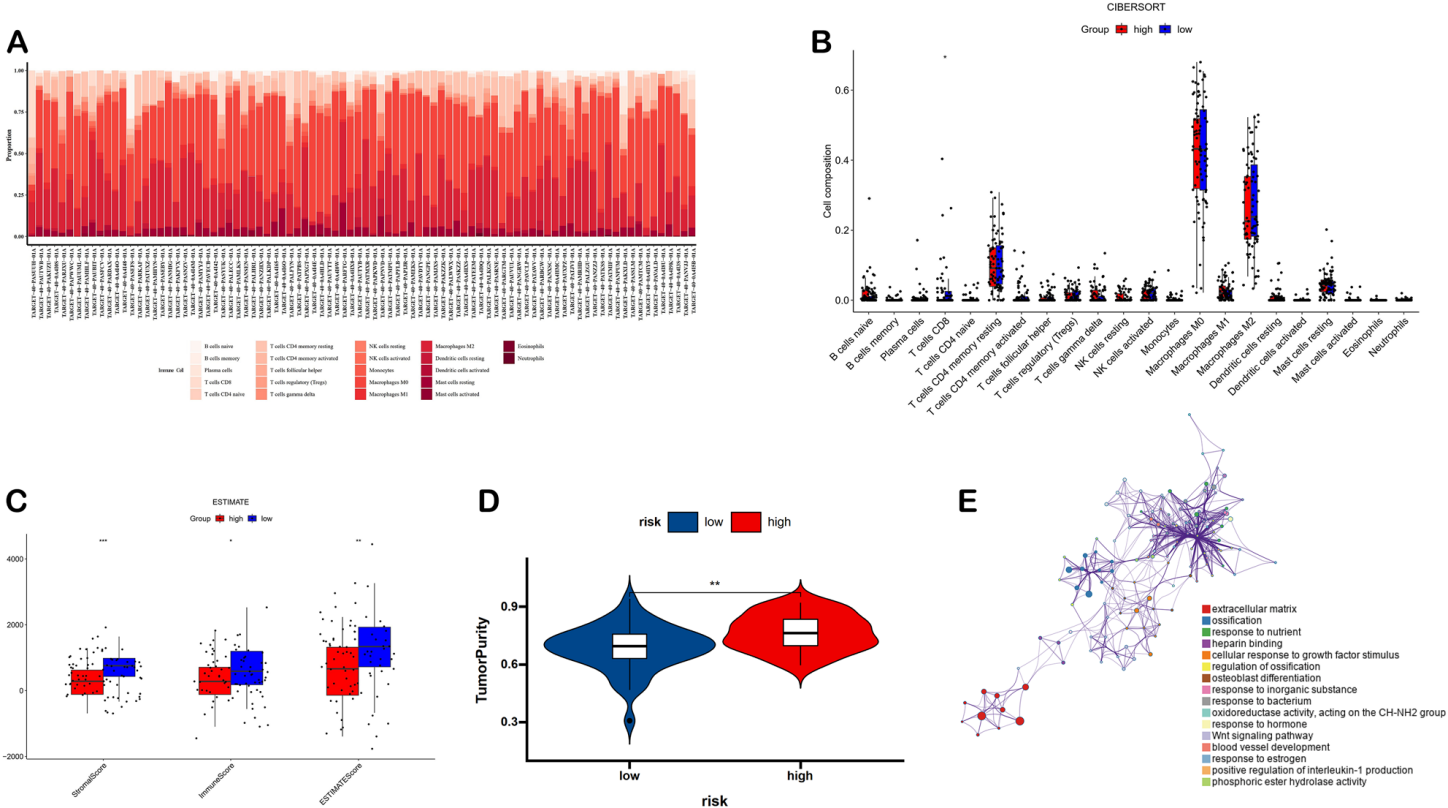
Immune infiltration and functional enrichment of DEGs between high risk and low risk group (**A**,**B**) CIBERSORT. (**C**,**D**) ESTIMATE. (**E**) Functional enrichment.

**Figure 4:**
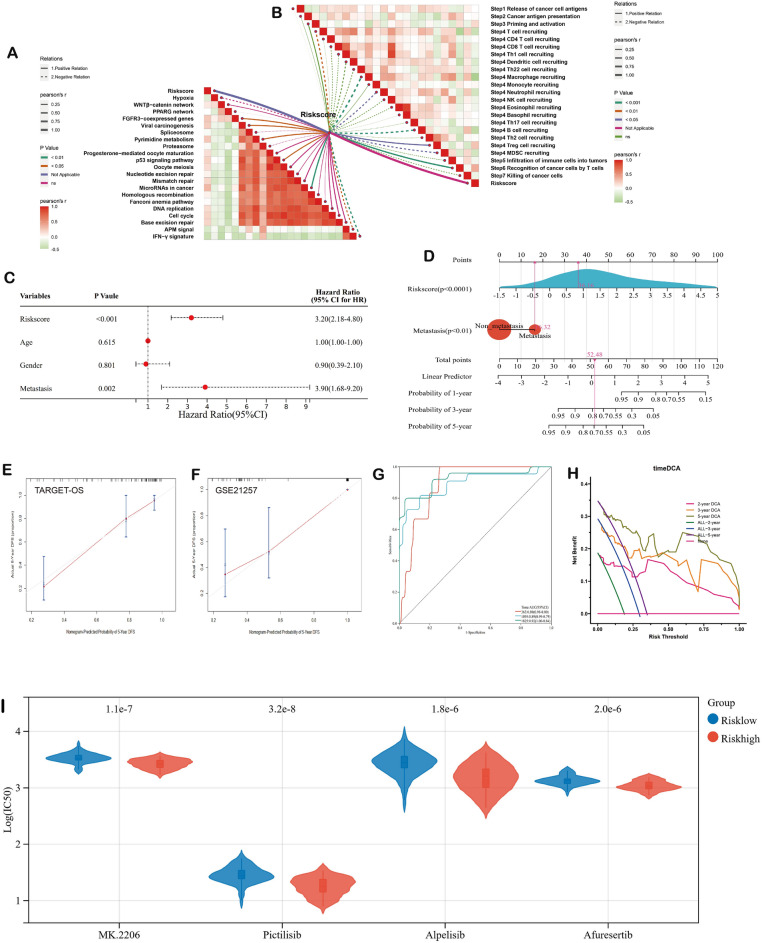
Immune-related analysis of risk score and construction and validation of the integrated nomogram. (**A**) Cancer immunity cycles. (**B**) Immunotherapy-predicted pathways. (**C**,**D**) Nomogram was constructed based on CMRGs-based risk scores and metastasis. (**E**) 5-year calibration plots of the nomogram in TARGET-OS cohort. (**F**) 5-year calibration plots of the nomogram in GSE21257 cohort. (**G**) The timeROC curve of the nomogram. (**H**) The timeDCA of the nomogram. (**I**) Four potential drugs of PI3K/Akt pathway for the treatment of OS. *ROC* receiver operating characteristic curve, *DCA* decisions curve analysis.

To access the predictable ability of the prognostic model, we separated the OS patients into the high and low risk group based on the median risk score and found that the risk score exhibited an excellent distinguishing ability between high and low risk groups in both TARGET-OS and GSE21257 datasets (Fig. 2A,B,D,E). Additionally, the timeROC curve signified the excellent accuracy of the prognostic model in both TARGET-OS and GSE21257 datasets (Fig. 2C,F). To explore the mechanism of our risk score model in OS patients, we perform the immune infiltration analysis and the enrichment analyses of the DEGs (Table S5) between high and low risk groups. For the immune infiltration analysis, the CD8 T cells in low risk group were found statistically higher than that of the high risk group in the CIBERSORT algorithm and the immune score of low risk group exhibited statistically higher than that of the high risk group in the ESTIMATE algorithm (Fig. 3A–D). For the enrichment analysis of the DEGs, the results were enriched in the extracellular matrix related pathways and response to signaling related pathways, suggesting the differential pathway activities between high- and low-risk groups (Fig. 3E, Table S6). Additionally, the analysis of cancer immunity cycles show that risk score is negative associated with T and B cells recruiting but positive associated with Treg recruiting. Furthermore, the analysis of immunotherapy-predicted pathways signified that risk score was correlated with the classical pathways including FGFR3-coexpressed genes, viral carcinogenesis, pyrimidine metabolism, p53 signaling pathway, microRNAs in cancer, APM signal, and IFN-γ signature (Fig. 4A,B, Tables S7, 8). Therefore, the risk score may be associated with the immune infiltration and signaling pathway activity, resulting in the different result for OS patients.

### Construction of the integrated nomogram

To establish a robust quantitative prognostic model for OS patients, we conducted an extensive multivariate Cox regression analysis, incorporating the risk score and essential clinical traits, including age, gender, and metastasis. The results of the multivariate Cox regression analysis revealed significant negative associations between the risk score [p value < 0.001; hazard ratio (HR) 3.20, 95% confidence interval (CI) 2.18–4.80] and metastasis [p value = 0.002; HR 3.90, 95% CI 1.68–9.20] with OS patient survival. Subsequently, these critical prognostic factors were thoughtfully integrated into the development of the nomogram (Fig. 4C,D). To assess the nomogram's predictive performance rigorously, we meticulously evaluated its calibration over a 5-year period and analyzed its timeROC curve, which unequivocally demonstrated its exceptional accuracy in predicting the prognosis of OS patients (Fig. 4E–G). Additionally, the timeDCA curve provided further validation of the remarkable clinical utility of our nomogram (Fig. 4H). Based on these compelling findings, our integrated nomogram emerges as a powerful and indispensable tool for clinicians, empowering them with valuable insights for treatment planning and reliable prognostic assessment of OS patients.

### Drugs with potential efficacy in OS

The Genomics of Drug Sensitivity in Cancer (GDSC), developed by the esteemed Sanger Institute in the UK, currently stands as the premier public resource for comprehensively assessing drug sensitivity in tumor cells. This repository consolidates invaluable data on tumor cell responses to diverse drugs, making it a critical asset in contemporary oncology research. To delineate potential small molecules that target CMRGs and enhance the clinical utility of our prognostic model, we undertook an in-depth drug sensitivity analysis. This analysis entailed a rigorous comparison of IC50 values between high- and low-risk groups. The results yielded significant insights, with MK.2206 (p = 1.1 × 10^7^), Pictilisib (p = 3.2 × 10^8^), Alpelisib (p = 1.8 × 10^6^), and Afuresertib (p = 2.0 × 10^6^) exhibiting substantial sensitivity in both high- and low-risk groups. Notably, patients classified within the high-risk group displayed heightened sensitivity to these drugs in comparison to their low-risk counterparts (Fig. 4I). These compelling findings hold immense therapeutic potential, potentially unveiling novel treatment avenues for OS patients. Leveraging the comprehensive data from GDSC and conducting a thorough drug sensitivity analysis have unveiled promising prospects for targeted therapeutic interventions, expanding the horizon of treatment possibilities for OS patients.

### Identification and validation of the independent prognostic CMRGs

**Figure 5:**
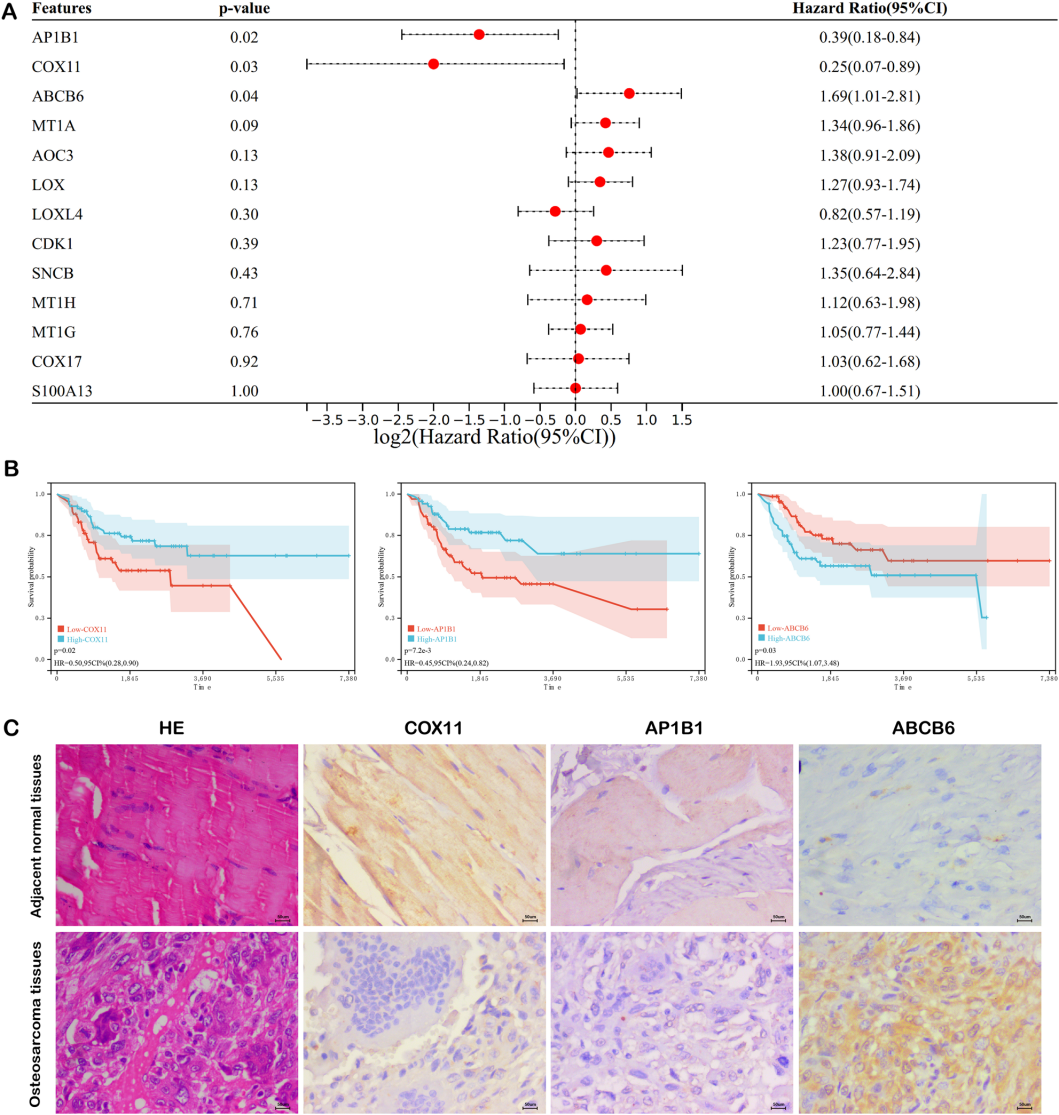
Expression validation of CMRGs (**A**) Multivariate Cox regression analysis of prognostic CMRGs. (**B**) Kaplan–Meier plot of COX11, AP1B1, and ABCB6. (**C**) Immunohistochemical staining of COX11, AP1B1, and ABCB6.

To identify independent prognostic genes for OS, we conducted multivariate Cox regression analysis encompassing all the genes included in the prognostic model. As a result, three genes, namely COX11, AP1B1, and ABCB6, were identified as independent prognostic signatures with p values less than 0.05 (Fig. 5A,B). Subsequently, we employed IHC to validate the differential expression of these independent prognostic signatures between OS tissues and adjacent normal tissues. Interestingly, the IHC results demonstrated that COX11 and AP1B1 expression levels were lower, while ABCB6 expression was significantly higher in OS tissue compared to adjacent normal tissue, aligning consistently with our bioinformatics findings (Fig. 5C). Given these findings, COX11, AP1B1, and ABCB6 hold the potential to serve as promising targets for OS treatment.

## Discussion

Over the past decades, with the introducion of multi-agent chemotherapy, neoadjuvant chemotherapy and radiotherapy, the long-term survival rate of OS patients have exciting improved^39^. Howerer, the extremely malignancy, highly tendentious invasion and metastasis, and resistance to traditional chemotherapy of OS cells have made the 5-year survival rate staggered at 60–70%^40^, posing clinical challenges for their treatment. Generally speaking, the reasons why the therapeutic effect of OS fail to reach that of other solid tumor can be summarized as absence of the sensitive classification index for patients with poor prognosis, the superficial understanding of OS immune microenvironment, and the genomic instability and complexity of OS^41^. Therefore, giving a profound insight into the molecule pathomechanism in term of the oncogenesis and progression of OS could help us to identify the specific biomarkers, making the early diagnosis, targeting therapy, and prognosis analysis of OS possible.

Recent, Science announced a novel type of cell death-cuproptosis, indicating the important role of copper in biological and pathological process^5,8^. The mounting evidence has underscored the significance of copper in biological and pathological processes, particularly in terms of cell death^42^, triggering our interests in investigating the role of copper metabolism during the pathological processes of OS. Hence, we formulated a hypothesis proposing that copper metabolism may exert a critical influence on the oncogenesis and progression of OS. Excitingly, the field of bioinformatics has undergone remarkable advancements, enabling profound insights into genetic markers and their implications in various diseases^43,44^. Meantime, theoretical modeling of protein signaling networks have provided new directions for the exploration of therapeutic targets^45,46^. Leveraging these advancements, bioinformatics has provided a myriad of sophisticated methodologies and diverse approaches to conduct in-depth investigations of these diseases, thereby enriching our comprehension of their underlying mechanisms and potential therapeutic targets^47^. To explore the role of copper metabolism in the pathological processes of OS, we embarked on an extensive exploration, utilizing comprehensive bioinformatics analysis. Initially, to assess the impact of copper metabolism in OS, we performed NMF clustering on the cohort of OS patients based on CMRGs obtained from the KEGG database. This analysis revealed a correlation between copper metabolism, prognosis, and the immune microenvironment of OS, hinting at its potential importance in the progression of the disease. Then, we utilized LASSO-COX regression analysis to construct the prognostic model including MT1A, MT1H, S100A13, CDK1, COX11, COX17, LOXL4, AOC3, MT1G, ABCB6, LOX, AP1B1, and SNCB, which are closely associated with copper metabolism and found the corresponding risk score calculated from the established model was associated with the prognosis and immune situation of OS. More importantly, the further analyses found that COX11, AP1B1, and ABCB6 might serve as the independent prognostic signatures of OS patients, offering potential therapeutic targets for OS treatment.

Over the decades, the increasing researches have witnessed the anti-cancer or/and cancer-promoting activity of the tumor immune microenvironment which exhibits a promising therapeutic direction^48–50^. In our studies, the functional enrichment including GO, KEGG, and GSEA analyses between the clusters were mainly related to the immune process. Additionally, ESIMATE algorithm shown that the stromalscore, immunescore, and estimatescore of cluster exhibiting a better prognosis is higher than that of cluster with a worse prognosis while the tumor purity shown an opposite trend. Therefore, copper metabolism may influence the TME of OS, triggering the markedly different prognosis for OS patients. As is widely known, tumor cells strengthen themselves by interacting with the component of the tumor microenvironment, which weakens the anti-tumor factors and intensify the tumor-promoting factors^39,51^. Previous studies have proved that NK cells, CD8+ T cells and B cells exert an anti-tumor effect while Tregs, TAMs, and DCs play a tumor-promoting role^39,52–54^. CIBERSORT in our studies also supported that compared with high-risk group, CD8+ T cells is higher in low-risk group, exerting anti-tumor effect. Additionally, the analysis of cancer immunity cycles show that risk score is negative associated with T and B cells recruiting but positive associated with Treg recruiting^37^. For a long time, the inadequate understanding of the immune microenvironment of OS resulted in the immunotherapy far from satisfactory^41^. Thus, we explored the relationship between our risk scores and certain canonical immunotherapeutic pathway to identify the potential target pathway for the treatment of OS. Excitingly, the analysis of immunotherapy-predicted pathways signified that risk score was correlated with the classical pathways including FGFR3-coexpressed genes, viral carcinogenesis, pyrimidine metabolism, p53 signaling pathway, microRNAs in cancer, APM signal, and IFN-γ signature. Therefore, our risk model exerted an excellent ability to predict the immune situation of TME in OS, offering a novel insight into the immunotherapy of OS.

Clinically, the clinical traits including location and size of tumor, chemotherapeutic effect, metastasis, and age are traditionally utilized to predict the prognosis of OS patients, failing to meet the satisfaction of accuracy^39,55^. To quantitatively predict the prognosis of OS patients and improve the accuracy of the risk stratification, we constructed an integrated nomogram via combining the CMRGs with metastasis. The calibration plots, timeROC curve, and timeROC curve signified the nomogram possessed an excellent clinical usability in term of survival prediction. Therefore, our studies have established a CMRGs-related risk model and nomogram, contributing to prognostic judgement and decision-making for clinician.

At present, treatments for OS patients mainly attribute to surgery in combined with chemotherapy, neoadjuvant chemotherapy and radiotherapy, which have immensely improved the long-term survival of OS patients^2^. However, the survival of patients developing chemotherapeutic resistance is still very poor and the severe adverse effects including high toxicity and the considerable risk of secondary disease sometime are life-threatening for OS patients, signifying the urgency to find new targets for the treatment of OS^56,57^. In our study, we ulitized GDSC database to perform the drug sensitivity analysis between high- and low-risk group and found high-risk group were more sensitive to four drug including Afuresertib, Alpelisib, Pictilisib, MK-2206, in which Afuresertib and MK-2206 are two inhibitors of Akt molecules whereas Alpelisib and Pictilisib are two inhibitors of phosphatidylinositol 3-kinase. As we known, PI3K/Akt pathway is consideres as one of the most canonial oncogenic pathways in various human cancers^58^. Similarly to other cancers, substantial evidence have demostrated the hyperactivity of PI3K/Akt pathway frequently occurs in OS and results in the pathological process of OS including tumorigenesis, proliferation, invasion, cell cycle progression, inhibition of apoptosis, angiogenesis, metastasis and chemotherapeutic resistance^58,59^. Furthermore, small molecule compounds blocking this pathway may present a promising potential therapy for OS^60–62^. Therefore, our study further confirmed the previously reported studies and highlighted the therapeutic potential of PI3K/Akt for the treatment of OS.

Finally, to explore the independent prognostic factors of OS patients, we employed multivariate Cox regression analysis for all prognostic CMRGs and identified COX11, AP1B1, and ABCB6 as three independent prognostic signatures. Further, we performed IHC to validate the differential expression of these three signatures between OS tissue and adjacent normal tissue. From the results of IHC, we found COX11 and AP1B1 exhibited low expression while ABCB6 was significantly up-regulated in OS tissue, while was consistent with our bioinformatics analysis. Interestingly, previous studies have highlighted the association between these three signatures (COX11, AP1B1, and ABCB6) and copper metabolism or cuproptosis^8,63,64^. COX11 (Cytochrome C Oxidase Copper Chaperone COX11) has been identified as copper donors to the Cu(B) sites of cytochrome oxidase, playing an important role in the mitochondrial respiratory chain^65^. AP1B1 (Adaptor Related Protein Complex 1 Subunit Beta 1) plays a role in protein sorting and has been found to disturb trafficking of copper transporters^64^. ABCB6 (ATP Binding Cassette Subfamily B Member 6) has been proved to conferred tolerance toward copper in rat^63^. The abnormal expression of these signatures are associated with the development of certain diseases including cancers^66–71^. Therefore, COX11, AP1B1, and ABCB6 can serve as the potential targets for OS treatment.

Undoubtedly, the present study is subject to several acknowledged limitations that warrant consideration. Firstly, the rare incidence of OS challenges in recruiting a large and diverse cohort for comprehensive analysis, potentially limiting the generalizability of the findings. Future investigations should aim to collaborate with multiple research centers or utilize international databases to overcome this limitation and enhance the robustness of the results. Secondly, it is imperative to recognize that the RNA-Seq and clinical data employed in this study predominantly originate from the TCGA and GEO databases, which primarily represent European and North American populations^72^. Consequently, inherent selection bias may exist, potentially limiting the applicability of the findings to other ethnic populations. Thus, validation of the results in diverse cohorts that encompass different ethnic backgrounds and geographical regions is essential to bolster the clinical relevance and broad utility of the findings. Lastly, while this study has provided valuable insights into the role of CMRGs in OS through bioinformatics analysis and validation of the CMRGs expression, the profound exploration of gene/protein signaling networks will be of paramount significance in advancing our understanding of regulatory mechanisms and identifying potential therapeutic targets in diseases^47,73^. Consequently, in our further work, functional assays and in vivo experiments are imperative to validate the potential therapeutic targets. This translational approach is indispensable to bridge the gap between preclinical research and clinical application, ultimately guiding the development of targeted therapeutic interventions for OS patients.

## Conclusion

In summary, this study innovatively described the role of copper metabolism in OS and systematically explored the expression pattern, prognostic value, and tumor immune microenvironment of CMRGs. Furthermore, we identified COX11, AP1B1, and ABCB6 as the potential targets for OS treatment, providing researchers and clinicians with new insight in early diagnosis and therapy of OS.

### Supplementary Information

Supplementary Information.

## Data Availability

The datasets supporting our findings are presented in the article.

## Abbreviations

OS: Osteosarcoma
CMRGs: Copper metabolism related genes
GEO: Gene Expression Omnibus
GO: Gene ontology
KEGG: Kyoto Encyclopedia of Genes and Genomes
NMF: Nonnegative matrix factorization
GSEA: Gene set enrichment analysis
ESTIMATE: Estimation of Stromal and Immune cells in Malignant Tumor tissues using Expression data
LASSO: Least absolute shrinkage and selection operator
DEGs: Differentially expressed genes
CIBERSORT: Cell type identification by estimating relative subsets of RNA transcripts

## References

[1] Wylie J (2004). Pathology and genetics of tumours of soft tissue and bone. Surg. Oncol..

[2] Roberts RD, Wedekind MF, Setty BA (2015). Chemotherapy Regimens for Patients with Newly Diagnosed Malignant Bone Tumors.

[3] Kaste SC, Pratt CB, Cain AM, Jones-Wallace DJ, Rao BN (1999). Metastases detected at the time of diagnosis of primary pediatric extremity osteosarcoma at diagnosis: Imaging features. Cancer.

[4] Wu CC, Livingston JA (2020). Genomics and the immune landscape of osteosarcoma. Adv. Exp. Med. Biol..

[5] Tsvetkov P (2022). Copper induces cell death by targeting lipoylated TCA cycle proteins. Science (New York, NY).

[6] Ruiz LM, Libedinsky A, Elorza AA (2021). Role of copper on mitochondrial function and metabolism. Front. Mol. Biosci..

[7] Brewer GJ (2003). Copper in medicine. Curr. Opin. Chem. Biol..

[8] Xue Q (2023). Copper metabolism in cell death and autophagy. Autophagy.

[9] Oliveri V (2022). Selective targeting of cancer cells by copper ionophores: An overview. Front. Mol. Biosci..

[10] Ishida S, Andreux P, Poitry-Yamate C, Auwerx J, Hanahan D (2013). Bioavailable copper modulates oxidative phosphorylation and growth of tumors. Proc. Natl. Acad. Sci. USA.

[11] Shanbhag VC (2021). Copper metabolism as a unique vulnerability in cancer. Biochim. Biophys. Acta Mol. Cell Res..

[12] Lelièvre P, Sancey L, Coll JL, Deniaud A, Busser B (2020). The multifaceted roles of copper in cancer: A trace metal element with dysregulated metabolism, but also a target or a bullet for therapy. Cancers.

[13] Harro CC, Smedley RC, Buchweitz JP, Langlois DK (2019). Hepatic copper and other trace mineral concentrations in dogs with hepatocellular carcinoma. J. Vet. Intern. Med..

[14] Lener MR (2016). Serum concentrations of selenium and copper in patients diagnosed with pancreatic cancer. Cancer Res. Treat..

[15] Safi R (2014). Copper signaling axis as a target for prostate cancer therapeutics. Can. Res..

[16] Aubert L, Nandagopal N, Roux PP (2020). Targeting copper metabolism to defeat KRAS-driven colorectal cancer. Mol. Cell. Oncol..

[17] Cui L (2021). Mitochondrial copper depletion suppresses triple-negative breast cancer in mice. Nat. Biotechnol..

[18] Liao Y (2020). Inflammation mobilizes copper metabolism to promote colon tumorigenesis via an IL-17-STEAP4-XIAP axis. Nat. Commun..

[19] Shao S, Si J, Shen Y (2019). Copper as the target for anticancer. Nanomedicine.

[20] Lelièvre P, Sancey L, Coll JL, Deniaud A, Busser B (2020). The multifaceted roles of copper in cancer: A trace metal element with dysregulated metabolism, but also a target or a bullet for therapy. Cancers.

[21] Li H (2020). The combination of disulfiram and copper for cancer treatment. Drug Discov. Today.

[22] da Silva DA (2022). Copper in tumors and the use of copper-based compounds in cancer treatment. J. Inorg. Biochem..

[23] Shanbhag V (2019). ATP7A delivers copper to the lysyl oxidase family of enzymes and promotes tumorigenesis and metastasis. Proc. Natl. Acad. Sci. USA.

[24] Milacic V, Jiao P, Zhang B, Yan B, Dou QP (2009). Novel 8-hydroxylquinoline analogs induce copper-dependent proteasome inhibition and cell death in human breast cancer cells. Int. J. Oncol..

[25] Goldman MJ (2020). Visualizing and interpreting cancer genomics data via the Xena platform. Nat. Biotechnol..

[26] Barrett T (2013). NCBI GEO: Archive for functional genomics data sets–update. Nucleic Acids Res..

[27] Buddingh EP (2011). Tumor-infiltrating macrophages are associated with metastasis suppression in high-grade osteosarcoma: A rationale for treatment with macrophage activating agents. Clin. Cancer Res..

[28] Kanehisa M, Goto S (2000). KEGG: Kyoto encyclopedia of genes and genomes. Nucleic Acids Res..

[29] Taminau J (2012). Unlocking the potential of publicly available microarray data using inSilicoDb and inSilicoMerging R/Bioconductor packages. BMC Bioinform..

[30] Johnson WE, Li C, Rabinovic A (2007). Adjusting batch effects in microarray expression data using empirical Bayes methods. Biostatistics (Oxford, England).

[31] Gene Ontology Consortium (2015). going forward. Nucleic Acids Res..

[32] Khatri P, Sirota M, Ten Butte AJ (2012). Years of pathway analysis: Current approaches and outstanding challenges. PLoS Comput. Biol..

[33] Subramanian A (2005). Gene set enrichment analysis: A knowledge-based approach for interpreting genome-wide expression profiles. Proc. Natl. Acad. Sci. USA.

[34] Yoshihara K (2013). Inferring tumour purity and stromal and immune cell admixture from expression data. Nat. Commun..

[35] Tibshirani R (1997). The lasso method for variable selection in the Cox model. Stat. Med..

[36] Newman AM (2015). Robust enumeration of cell subsets from tissue expression profiles. Nat. Methods.

[37] Xu L (2018). TIP: A web server for resolving tumor immunophenotype profiling. Can. Res..

[38] Hu J (2021). Siglec15 shapes a non-inflamed tumor microenvironment and predicts the molecular subtype in bladder cancer. Theranostics.

[39] Gill J, Gorlick R (2021). Advancing therapy for osteosarcoma. Nat. Rev. Clin. Oncol..

[40] Ritter J, Bielack SS (2010). Osteosarcoma. Ann. Oncol..

[41] Meltzer PS, Helman LJ (2021). New horizons in the treatment of osteosarcoma. N. Engl. J. Med..

[42] Xue Q (2023). Copper metabolism in cell death and autophagy. Autophagy.

[43] Wang T, Sun J, Zhao Q (2023). Investigating cardiotoxicity related with hERG channel blockers using molecular fingerprints and graph attention mechanism. Comput. Biol. Med..

[44] Wang W, Zhang L, Sun J, Zhao Q, Shuai J (2022). Predicting the potential human lncRNA-miRNA interactions based on graph convolution network with conditional random field. Brief. Bioinform..

[45] Li X (2021). RIP1-dependent linear and nonlinear recruitments of caspase-8 and RIP3 respectively to necrosome specify distinct cell death outcomes. Protein Cell.

[46] Xu F (2023). Specificity and competition of mRNAs dominate droplet pattern in protein phase separation. Phys. Rev. Res..

[47] Sun F, Sun J, Zhao Q (2022). A deep learning method for predicting metabolite-disease associations via graph neural network. Brief. Bioinform..

[48] Gajewski TF, Schreiber H, Fu YX (2013). Innate and adaptive immune cells in the tumor microenvironment. Nat. Immunol..

[49] Bagaev A (2021). Conserved pan-cancer microenvironment subtypes predict response to immunotherapy. Cancer Cell.

[50] Zhou Y (2020). Single-cell RNA landscape of intratumoral heterogeneity and immunosuppressive microenvironment in advanced osteosarcoma. Nat. Commun..

[51] Hinshaw DC, Shevde LA (2019). The tumor microenvironment innately modulates cancer progression. Can. Res..

[52] Wang X (2021). Identification of an immune-related signature indicating the dedifferentiation of thyroid cells. Cancer Cell Int..

[53] Chen C (2021). Immunotherapy for osteosarcoma: Fundamental mechanism, rationale, and recent breakthroughs. Cancer Lett..

[54] Cersosimo F (2020). Tumor-associated macrophages in osteosarcoma: From mechanisms to therapy. Int. J. Mol. Sci..

[55] Bieling P (1996). Tumor size and prognosis in aggressively treated osteosarcoma. J. Clin. Oncol..

[56] Longhi A (2012). Late effects of chemotherapy and radiotherapy in osteosarcoma and Ewing sarcoma patients: The Italian Sarcoma Group Experience (1983–2006). Cancer.

[57] Lilienthal I, Herold N (2020). Targeting molecular mechanisms underlying treatment efficacy and resistance in osteosarcoma: A review of current and future strategies. Int. J. Mol. Sci..

[58] Zhang J, Yu XH, Yan YG, Wang C, Wang WJ (2015). PI3K/Akt signaling in osteosarcoma. Clin. Chim. Acta Int. J. Clin. Chem..

[59] Díaz-Montero CM, Wygant JN, McIntyre BW (2006). PI3-K/Akt-mediated anoikis resistance of human osteosarcoma cells requires Src activation. Eur. J. Cancer (Oxford, England: 1990).

[60] Chen C (2022). PI3K inhibitor impairs tumor progression and enhances sensitivity to anlotinib in anlotinib-resistant osteosarcoma. Cancer Lett..

[61] Inoue R (2005). The inhibitory effect of alendronate, a nitrogen-containing bisphosphonate on the PI3K-Akt-NFkappaB pathway in osteosarcoma cells. Br. J. Pharmacol..

[62] Chen J (2013). Dipsacus asperoides polysaccharide induces apoptosis in osteosarcoma cells by modulating the PI3K/Akt pathway. Carbohyd. Polym..

[63] Jalil YA (2008). Vesicular localization of the rat ATP-binding cassette half-transporter rAbcb6. Am. J. Physiol. Cell Physiol..

[64] Alsaif HS (2019). Homozygous loss-of-function mutations in AP1B1, encoding beta-1 subunit of adaptor-related protein complex 1, cause MEDNIK-like syndrome. Am. J. Hum. Genet..

[65] Horng YC, Cobine PA, Maxfield AB, Carr HS, Winge DR (2004). Specific copper transfer from the Cox17 metallochaperone to both Sco1 and Cox11 in the assembly of yeast cytochrome C oxidase. J. Biol. Chem..

[66] Tang L (2012). Association of STXBP4/COX11 rs6504950 (G>A) polymorphism with breast cancer risk: Evidence from 17,960 cases and 22,713 controls. Arch. Med. Res..

[67] Barresi V (2016). Transcriptome analysis of copper homeostasis genes reveals coordinated upregulation of SLC31A1, SCO1, and COX11 in colorectal cancer. FEBS Open Bio.

[68] Ahmed S (2009). Newly discovered breast cancer susceptibility loci on 3p24 and 17q23.2. Nat. Genet..

[69] Decock A (2022). mRNA capture sequencing and RT-qPCR for the detection of pathognomonic, novel, and secondary fusion transcripts in FFPE tissue: A sarcoma showcase. Int. J. Mol. Sci..

[70] She Q (2022). ABCB6 knockdown suppresses melanogenesis through the GSK3-β/β-catenin signaling axis in human melanoma and melanocyte cell lines. J. Dermatol. Sci..

[71] Zhao SG (2013). Increased expression of ABCB6 enhances protoporphyrin IX accumulation and photodynamic effect in human glioma. Ann. Surg. Oncol..

[72] Tomczak K, Czerwińska P, Wiznerowicz M (2015). The Cancer Genome Atlas (TCGA): An immeasurable source of knowledge. Contemp. Oncol. (Poznan, Poland).

[73] Li X (2022). Caspase-1 and gasdermin D afford the optimal targets with distinct switching strategies in NLRP1b inflammasome-induced cell death. Research (Washington, DC).

