# Supplementary material for: Comprehensive analysis of copper-metabolism-related genes about prognosis and immune microenvironment in osteosarcoma
Source: Sci Rep. 2023 Sep 12;13:15059. doi: 10.1038/s41598-023-42053-w (PMC10497601; doi:10.1038/s41598-023-42053-w)
Supplement: Supplementary file 1 — Supplementary Information. [file 41598_2023_42053_MOESM1_ESM.zip › supplementary data/Supplementary figure.docx]

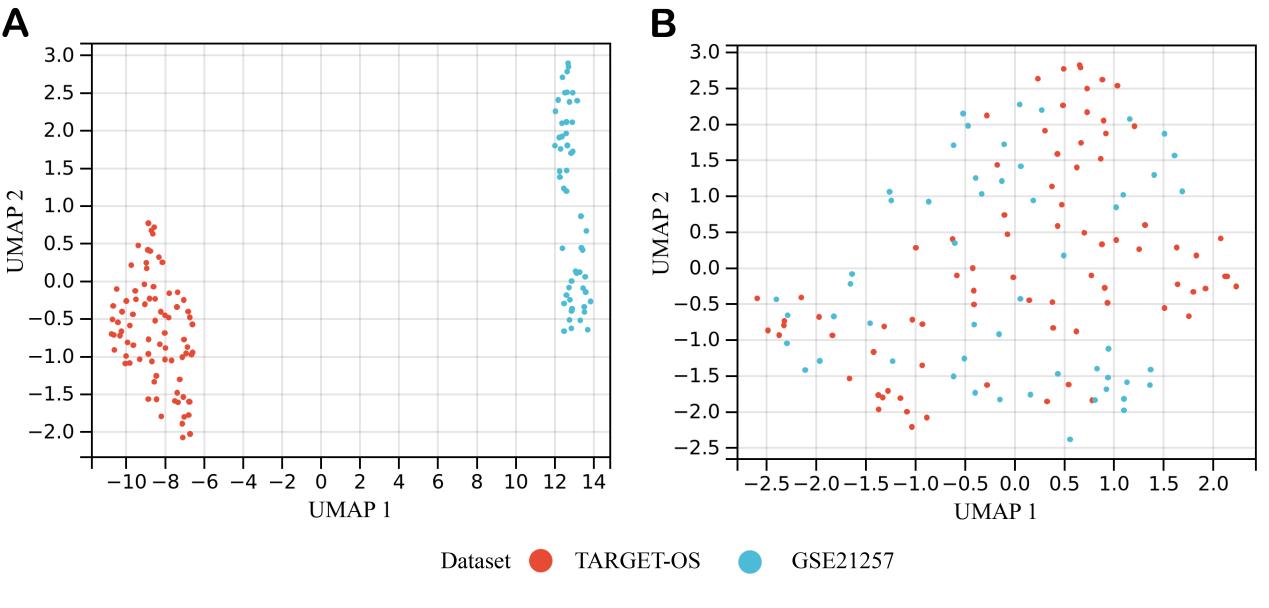


Figure S1 (A) Before batch effect removal. (B) After batch effect removal.


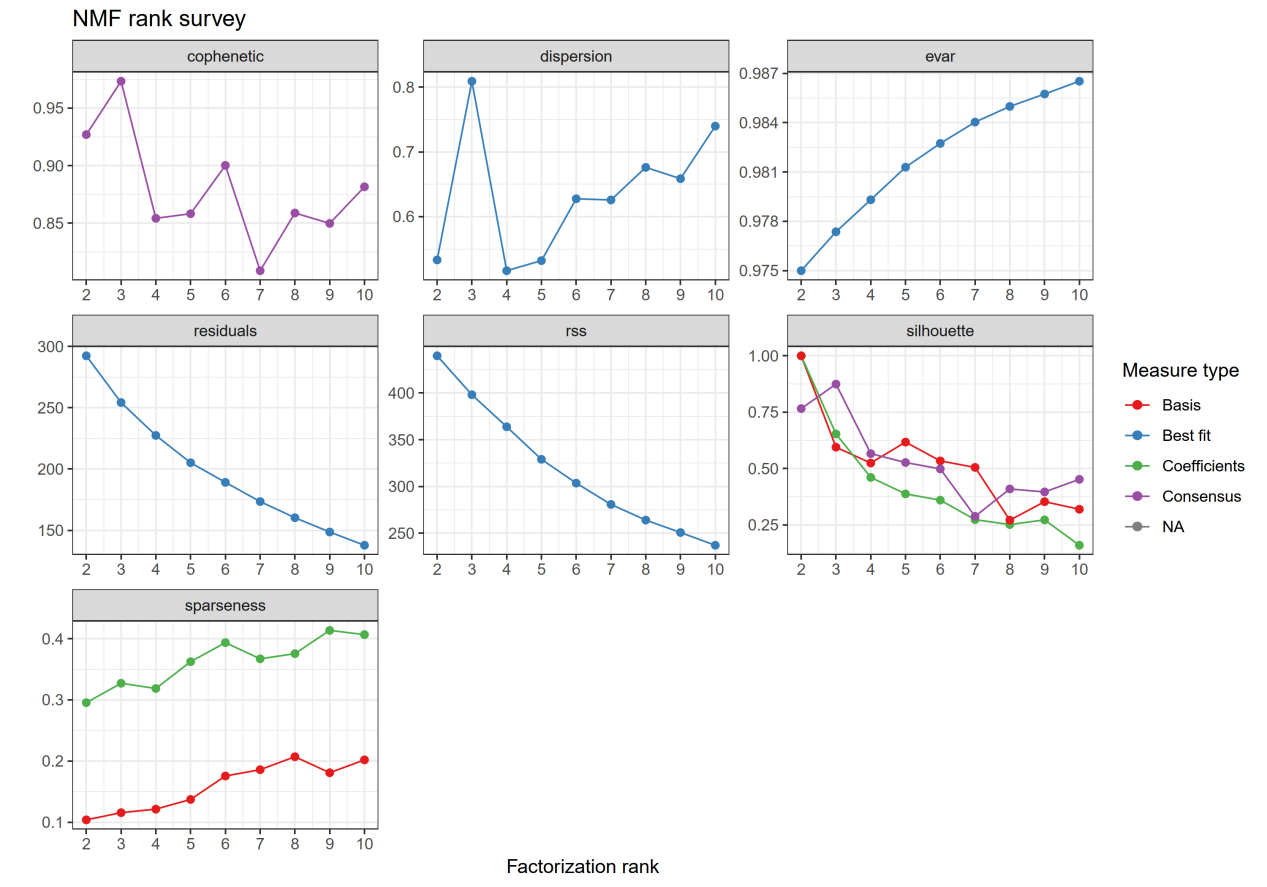


Figure S2 The screening process of molecular subgroups through nonnegative matrix factorization (NMF) cluster: the NMF rank survey results showing an optimal grouping of two subgroups


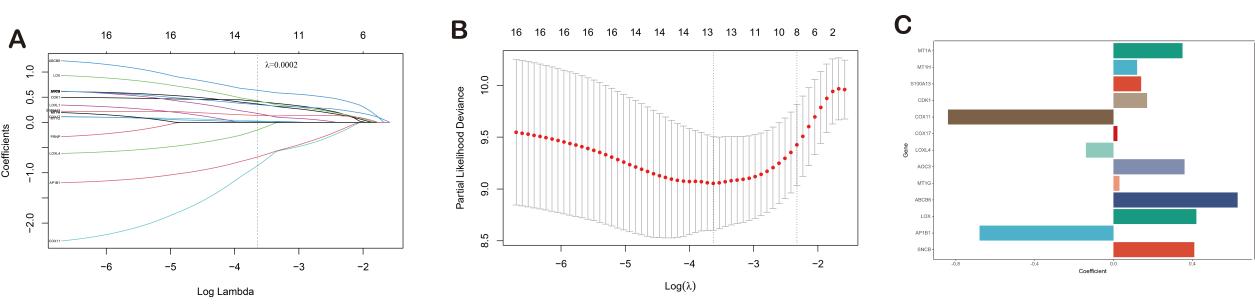


Figure S3 (A-C) LASSO-COX regression of prognosis-related CMRGs


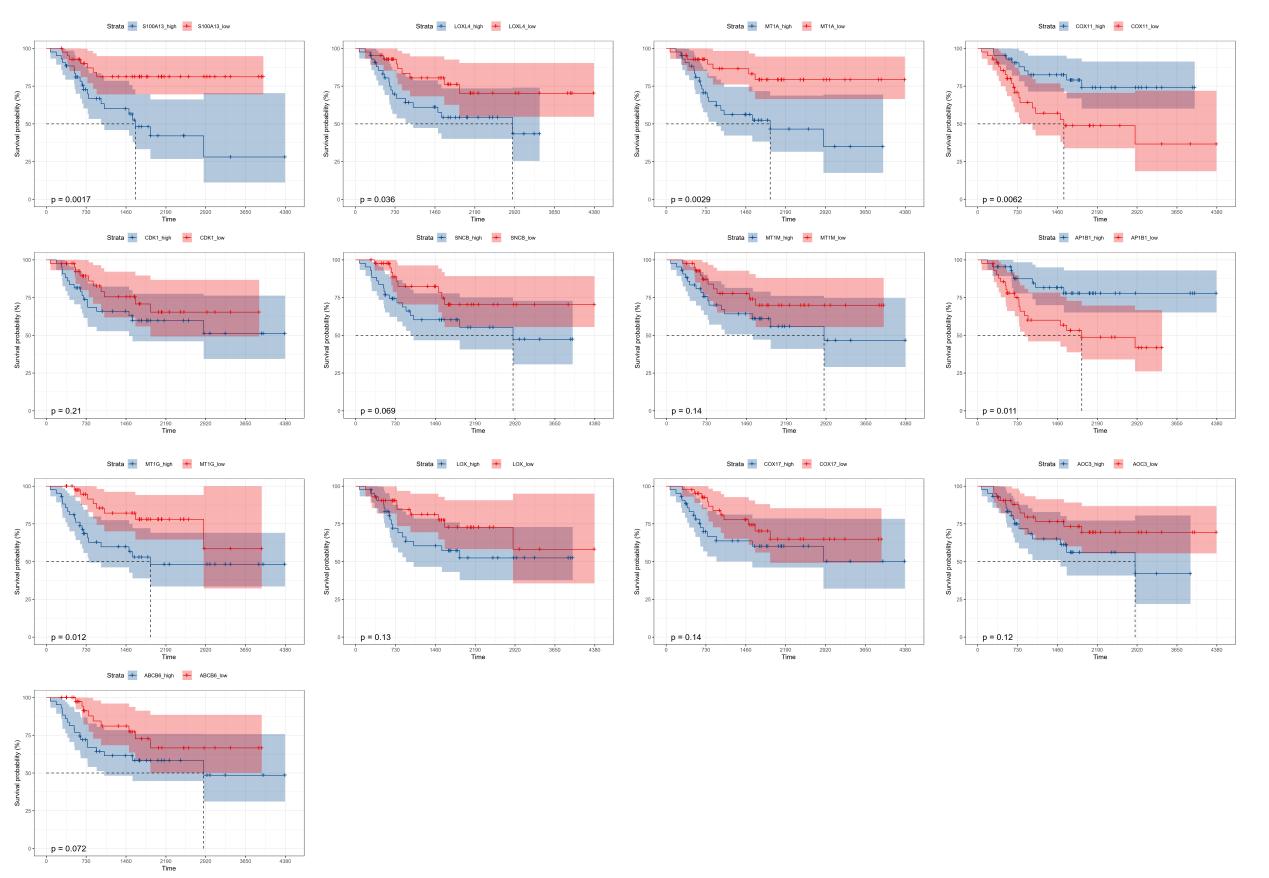


Figure S4 The survival curve of patients grouped by the expression level of 13 prognosis-related CMRGs used for model construction.
